# Further evaluation of recombinant Tsol-p27 by enzyme-linked immunoelectrotransfer blot for the serodiagnosis of cysticercosis in pigs from Mozambique

**DOI:** 10.1186/s13071-019-3816-x

**Published:** 2019-11-27

**Authors:** Noemia Nhancupe, Emilia V. Noormahomed, Sonia Afonso, Staffan Svard, Johan Lindh

**Affiliations:** 1 Department of Microbiology, Faculty of Medicine, Eduardo Mondlane University, Maputo, Mozambique; 2 Department of Cell and Molecular Biology, BMC, Uppsala University, Uppsala, Sweden; 3 Department of Medicine, Infectious Disease Division, University of California, San Diego, USA; 4 Mozambique Institute for Health Education and Research (MIHER), Maputo, Mozambique; 5 Department of Clinics, Veterinary Faculty, Eduardo Mondlane University, Maputo, Mozambique

**Keywords:** Recombinant Tsol-p27, *Taenia solium*, Porcine cysticercosis, Human cysticercosis, Immunodiagnostic, Enzyme-linked immunoelectrotransfer blot, Mozambique

## Abstract

**Background:**

Porcine cysticercosis has a negative impact on human health and the meat industry, as it makes infected meat unaproprieted for consuption and it is the main etiology of epileptic seizures in developing countries. There are multiple serological assays that use crude antigens with high sensitivity and specificity for the diagnosis of both porcine and human cysticercosis. Nonetheless, antigen preparation is time-consuming, needs a well-equipped laboratory and trained personnel and places those manipulating the meat at great risk for infection. New serodiagnostic approaches to the diagnosis of porcine and human cysticercosis have been directed towards the development of recombinant deoxyribonucleic acid technology for the generation of synthetic proteins that can serve as simplified, low-cost and harmless substitutes for native antigens. The aim of the present study was to further evaluate the recombinant Tsol-p27 protein as a target molecule in immunoassays for the serodiagnosis of porcine cysticercosis. From these data, we hoped to develop recommendations regarding its use in the serodiagnosis of porcine cysticercosis.

**Results:**

We studied a panel of 83 naturally infected pig sera from Angónia District, Mozambique, an endemic area for porcine and human cysticercosis. These sera were previously tested by antigen enzyme-linked immunosorbent assay (Ag-ELISA) to detect antigens of *T. solium*. The serum panel was processed by enzyme-linked immunoelectrotransfer blot (EITB) assay against the recombinant Tsol-p27 protein and the Ag-ELISA assay results were used to compare and evaluate the performance of Tsol-p27 for the diagnosis of cysticercosis. Out of 83 sera, 24 (29.0%) were positive for Tsol-p27 and 59 (71%) were negative in the same assay. From the 37 sera that tested positive to Ag-ELISA, 11 (13.3%) were positive to Tsol-p27, while from 46 sera that tested negative to Ag-ELISA, 33 (39.7%) also tested negative to Tsol-p27. The sensitivity and specificity of Tsol-p27 was 29.7% and 71.7%, respectively, while the positive predictive value and negative predictive value were 45.8% and 55.9%, respectively, as calculated using Medcalc® version 15.0 software (MedCalc Software, Ostend, Belgium).

**Conclusion:**

While Tsol-p27 recombinant protein might be suitable for testing human sera, its performance in pigs is not acceptable, so other recombinant proteins should be evaluated alone or multiplexed.

## Background

Cysticercosis, caused by the larvae of *Taenia solium* is a zoonotic and poverty-related disease that causes serious public health and agricultural problems in endemic countries of Africa, Latin America, and Asia [1–4]. It is estimated that between 80–90% of seizures and other neurological disorders in developing countries are due to infection with *T. solium* larva [1, 5]. Infected pig meat is inappropriate for consumption, resulting in substantial economical losses for the meat industry and small farmers. Because of this, the World Health Organization has called for action to either control or eliminate the disease, both in humans and in pigs [3].

Serology for antigen or antibody detection either in humans or in pigs, using total or partial *T*. *solium* extracts of the metacestode such as cyst fluid, scolex or extracts from external membranes, is frequently done using different preparations of *T*. *solium* metacestodes. The assays using these preparations have a sensitivity varying from 38.9% to 99% and a specificity ranging from 48.3% to 100% [1, 6–9]. However, the use of crude or purified antigens is time-consuming, needs a well-equipped laboratory, trained personnel and represents a significant risk of infection to those manipulating the infected meat [10–12].

New serodiagnostic approaches have been directed towards the development of recombinant deoxyribonucleic acid technology for generation of antigenic proteins to serve as simplified, low-cost substitutes for native antigens and without risk of infection to those preparing the antigen [10–12]. Tsol-p27 developed at Uppsala University and tested on human sera from Nicaragua and Mozambique [13, 14] is an example of this approach. The aim of the present study was to further evaluate the recombinant Tsol-p27 protein as a target molecule in immunoassays for the serodiagnosis of porcine cysticercosis [13–15]. From these data, we hoped to develop recommendations regarding its use in the serodiagnosis of porcine cysticercosis.

## Methods

### Pig serum samples

To evaluate the immunogenicity of Tsol-p27 recombinant protein for detection of antibodies to porcine cysticercosis, we used a panel of 83 serum samples from naturally infected pigs in Angónia District, Tete Province, Mozambique, an area endemic for porcine and human cysticercosis [16, 17]. Sera were stored at − 80 °C at the Veterinary Faculty of Eduardo Mondlane University, Mozambique, after processing for the detection of cysticercus antigens using the antigen enzyme-linked immunosorbent assay (Ag-ELISA) as described by Dorny et al. [18]. From the panel of 83 samples, 37 (44.6%) were positive and 46 (55.4%) were negative in the Ag-ELISA assay. In addition, as a positive control, we used sera from three pigs that demonstrated a large number of cysts at necropsy and were also positive for cysticercal antigen in the Ag-ELISA assay [16].

### Enzyme-linked immunoelectrotransfer blot analysis

Enzyme-linked immunoelectrotransfer blot (EITB) analysis was performed as described previously by our group with human sera [15], using the recombinant Tsol-p27 protein prepared according to Salazar-Anton et al. [14].

Briefly, recombinant Tsol-p27 protein was separated by 12% sodium dodecyl sulfate polyacrylamide gel and transferred to a nitrocellulose membrane. The membranes were blocked for 2 h in blocking solution (5% skimmed milk in phosphate-buffered saline), and then rinsed in washing buffer containing 0.005% Tween 20. Thereafter, the membranes were incubated for 3 h with pig sera diluted 1:500 in blocking solution and then for 1 h with rabbit anti-pig IgG conjugated with peroxidase (Sigma-Aldrich, Stockholm, Sweden) diluted 1:3000 in blocking solution. Detection was performed according to the embeddable common lisp plus manual (GE Healthcare, Uppsala, Sweden) [15]. A sample was considered positive if a band could be seen at 27 kD [13–15].

### Statistical analysis

Data were entered into Microsoft Office Excel 2010 datasheets. Statistical calculations of the frequencies were performed using STATA 10.0 software (StataCorp LP, Texas, USA).

Results of the the Ag-ELISA and EITB Tsol-p27 recombinant protein were compared and analysed to determine the specificity, sensitivity, positive predictive value (PPV) and negative predictive value (NPV) of the Tsol-p27 recombinant protein, and using the Medcalc® version 15.0 software (MedCalc Software, Ostend, Belgium).

## Results

Results of bivariate analysis are presented in Table 1. Out of the 83 sera studied, 24 (29.0%) were positive for Tsol-p27 and 59 (71.0%) were negative in the same assay. From the 37 sera that tested positive to Ag-ELISA, 11 (13.3%) were positive to Tsol-p27, while from 46 sera that tested negative to Ag-ELISA, 33 (39.7%) also tested negative to Tsol-p27. The recombinant Tsol-p27 assay for the diagnosis of cysticercosis resulted in a sensitivity of 29.7% and a specificity of 71.7% when compared to Ag-ELISA assay, respectively, while the positive predictive value and negative predictive value were 45.8% and 55.9%, respectively.

**Table 1:** Detection of antibodies against *T.solium* cysticerci in sera of some pigs from Angónia using EITB Tsol-p27 compared with Ag-ELISA assay

Serological results	EITB Tsol-p27		
	Positive *n* (%)	Negative *n* (%)	Total *n* (%)
Ag-ELISA-positive	11 (13.3)	26 (31.3)	37 (44.6)
Ag-ELISA-negative	13 (15.7)	33 (39.7)	46 (55.4)
Total	24 (29.0)	59 (71.0)	83 (100)

*Note*: 83 sera tested for Ag-ELISA assay and for EITB Tsol-p27

## Discussion

The aim of the present study was to further evaluate the recombinant Tsol-p27 antigen as a candidate for the serodiagnosis of porcine cysticercosis in an EITB assay to develop recommendations for its use in this setting.

Our assay achieved detection at a sensitivity of 29.7% and a specificity of 71.7%, which are both low when compared to previous studies using the same recombinant Tsol-p27 in human sera from Nicaragua and Mozambique. In these settings, the assay has been reported with a substantially greater sensitivity of 86.7% and a higher specificity of 97.8% [13, 14]. These levels are comparable to results obtained by other investigators using an EITB with one or seven bands to determine seropositivity [1, 8, 9].

The relatively low specificity observed in the samples in our assay can be explained by the influence of the environment, the pigs carrying different species of worms that give rise to a mixture of different antigens and antibody responses or even because pigs respond to different antigens from humans. Furthermore, the use of Ag-ELISA has been reported to be complicated by cross-reactivity with *Taenia hydatigena* larvae [1, 18]. The prevalence of *T. hydatigena* larvae in pigs in Africa is low (9.9%) [19], and it is thus not likely that such cross-reactivity was the cause of the low specificity we found in our assay.

Serological methods are crucial instruments to support the diagnosis of cysticercosis in animals with light or recent infections as well as in epidemiological studies [1, 5, 6, 10, 11]. The EITB assay using Tsol-p27 antigen can be a useful tool to diagnose swine cysticercosis in endemic countries such as Mozambique with limited resources [13–15].

Despite the low sensitivity and specificity of Tsol-p27 recombinant protein when compared Ag-ELISA assay, it can be mass produced, does not require use of live parasite material, is easy to perform and is an economical alternative for field studies, especially in developing countries.

However, two limitations of this study deserve to be highlighted. First, as this was an exploratory study, we tested a low number of sera samples. Secondly, the sensitivity and specificity of Tsol-p27 could not be compared to the gold standard test. Therefore the sensitivity and specificity of our assay could be higher or lower.

Future studies should be targeted for the use of EITB Tsol-p27 in combination with other tests or mixed proteins in order to improve the overall validity. These studies may increase the accuracy of results and provide better estimation of porcine cysticercosis infection in rural communities.

## Conclusions

While Tsol-p27 recombinant protein might be suitable for testing human sera against infection by the larva of *T. solium*, its performance in pigs sera is not acceptable, and other recombinant proteins should be evaluated alone or multiplexed.

## Data Availability

The datasets used during the present study are available from the corresponding author on reasonable request.

## Abbreviations

Ag-ELISA: antigen enzyme-linked immunosorbent assay
EITB: enzyme-linked immunoelectrotransfer blot
PPV: positive predictive value
NPV: negative predictive value
